# Elevated levels of autoantibodies against DNAJC2 in sera of patients with atherosclerotic diseases

**DOI:** 10.1016/j.heliyon.2020.e04661

**Published:** 2020-08-19

**Authors:** Yoichi Yoshida, Xiao-Meng Zhang, Hao Wang, Toshio Machida, Seiichiro Mine, Eiichi Kobayashi, Akihiko Adachi, Tomoo Matsutani, Ikuo Kamitsukasa, Takeshi Wada, Akiyo Aotsuka, Katsuro Iwase, Go Tomiyoshi, Rika Nakamura, Natsuko Shinmen, Hideyuki Kuroda, Hirotaka Takizawa, Koichi Kashiwado, Hideo Shin, Yuichi Akaogi, Junichiro Shimada, Eiichiro Nishi, Mikiko Ohno, Minoru Takemoto, Koutaro Yokote, Kenichiro Kitamura, Yasuo Iwadate, Takaki Hiwasa

**Affiliations:** a Department of Neurological Surgery, Graduate School of Medicine, Chiba University, Chiba 260-8670, Japan; b Department of Biochemistry and Genetics, Graduate School of Medicine, Chiba University, Chiba 260-8670, Japan; c Comprehensive Stroke Center, Chiba University Hospital, Chiba 260-8677, Japan; d Department of Anesthesia, The First Affiliated Hospital, Jinan University, Guangzhou 510632, PR China; e Department of Neurosurgery, Chiba Cerebral and Cardiovascular Center, Ichihara, 290-0512, Chiba, Japan; f Department of Neurosurgery, Eastern Chiba Medical Center, Chiba 283-8686, Japan; g Department of Neurosurgery, Sawara Prefectural Hospital, Chiba 287-0003, Japan; h Department of Neurology, Chiba Rosai Hospital, Chiba 290-0003, Japan; i Department of Neurology, Chibaken Saiseikai Narashino Hospital, Chiba 275-8580, Japan; j Department of Internal Medicine, Chiba Aoba Municipal Hospital, Chiba 260-0852, Japan; k Medical Project Division, Research Development Center, Fujikura Kasei Co., Saitama 340-0203, Japan; l Port Square Kashiwado Clinic, Kashiwado Memorial Foundation, Chiba 260-0025, Japan; m Department of Neurology, Kashiwado Hospital, Chiba 260-0854, Japan; n Department of Neurosurgery, Higashi Funabashi Hospital, Chiba 274-0065, Japan; o Department of Neurology, Chiba Cerebral and Cardiovascular Center, Chiba 290-0512, Japan; p Department of Cardiovascular Medicine, Graduate School of Medicine, Kyoto University, Kyoto 606-8507, Japan; q Department of Pharmacology, Shiga University of Medical Science, Shiga 520-2192, Japan; r Department of Clinical Cell Biology and Medicine, Graduate School of Medicine, Chiba University, Chiba 260-8670, Japan; s Department of Diabetes, Metabolism and Endocrinology, School of Medicine, International University of Health and Welfare, Chiba 286-8686, Japan; t Department of Internal Medicine 3, University of Yamanashi School of Medicine, Yamamashi 409-3898, Japan

**Keywords:** Cardiovascular system, Hematological system, Neurology, Neurosurgery, Clinical research, Diagnostics, Biomarkers, DNAJC2, Acute ischemic stroke, Acute myocardial infarction, Autoantibody biomarker, Atherosclerosis, Cardiology

## Abstract

**Background:**

Serum antibody markers have been increasingly identified not only for cancer and autoimmune diseases but also for atherosclerosis-related diseases such as acute ischemic stroke (AIS), acute myocardial infarction (AMI), diabetes mellitus (DM), and chronic kidney disease (CKD). Biomarkers for transient ischemic attack (TIA) and non-ST segment elevation acute coronary syndrome (NSTEACS) are potentially useful for detection of early phase of atherosclerotic changes against AIS and AMI, respectively.

**Methods:**

We utilized serological identification of antigens by recombinant cDNA expression cloning (SEREX) using a human aortic endothelial cell cDNA phage library and sera from patients with TIA or NSTEACS. Serum antibody levels were measured by amplified luminescent proximity homogeneous assay-linked immunosorbent assay (AlphaLISA) using purified recombinant antigens.

**Results:**

Screening of sera from patients with TIA identified DnaJ heat shock protein family (Hsp40) member C2 (DNAJC2) as a candidate antigen, which was also isolated by SEREX screening using sera of patients with NSTEACS. The validation cohort revealed significantly higher DNAJC2 antibody (DNAJC2-Ab) levels in the sera of patients with TIA or AIS than those in healthy donors (HDs). Multivariate logistic regression analysis indicated that the predictive odds ratios (OR) of DNAJC2-Ab levels for TIA and AIS were 2.54 (95% confidence interval [CI]: 1.36–4.74, *p* = 0.0034) and 2.14 (95% CI: 1.39–3.30, *p* = 0.0005), respectively. Serum DNAJC2-Ab levels were also higher in patients with AMI, DM, and CKD than those in HDs.

**Conclusion:**

Serum DNAJC2-Ab level may be useful for early detection of atherosclerotic lesions, which lead to AIS and AMI.

## 1 Introduction

To date, among the several factors that are recognized to underlie the progression of atherosclerosis are age, high blood pressure, hyperlipidemia, diabetes mellitus (DM), chronic kidney disease (CKD), smoking, and obesity [1]. The most serious consequences of atherosclerosis are acute ischemic stroke (AIS) and acute myocardial infarction (AMI), which frequently leads to sudden death, severe sequelae, and permanent disability. Early diagnosis and treatment of atherosclerosis are indispensable to prevent the onset of AIS and AMI. Many risk factors for atherosclerosis have been reported, including hypertension, hyperlipidemia, increased body mass index (BMI)/obesity, smoking, and family history [2, 3, 4]. Blood tests to measure high-density lipoprotein (HDL)-cholesterol, low-density lipoprotein (LDL)-cholesterol, total cholesterol (TC), glycohemoglobin (HbA1c) [5], and uric acid [6], which were introduced to evaluate atherosclerosis development, remain insufficient.

Conversely, atherosclerosis exhibits several characteristics of chronic inflammatory disease, and various immune cells have been reported to play roles in atherogenesis [7, 8, 9, 10, 11]. Several studies identified antigens associated with atherosclerotic diseases including oxidized low-density lipoprotein (oxLDL) [12], apolipoprotein A-1 [13], and β2-glycoprotein I for atherosclerosis [14]; phospholipids [15], nardilysin [16] and heat shock proteins (Hsps) for CVD [17]; Hsp60 for stroke [18]; and insulin [19], glutamic acid decarboxylase (GAD) [20], and protein tyrosine phosphatase IA-2 [21,22] for DM.

Serological identification of antigens by recombinant cDNA expression cloning (SEREX) is an excellent method for identifying antigenic proteins [23, 24]. We previously identified atherosclerosis-associated antibody markers, including those against RPA2 [25], SOSTDC1 [26], CBX1 [27], and PDCD11 [28] for AIS; CTNND1 [26] for CVD; TUBB2C [29], CCNG2 [26], GADD34 [30], and adiponectin [31] for DM; COPE [32] and NBL1 [33] for obstructive sleep apnea; and ATP2B4 [34], BMP-1 [25,34], DHPS [35], SH3BP5 [36] PRCP [37], and MMP1 [27] for common atherosclerotic diseases. Repeated leaking of antigenic proteins to the circulation due to tissue destruction during the progression of atherosclerosis could lead to a substantial increase in the levels of antibodies against these proteins. Immunoglobulin (Ig) G antibodies are highly stable in the circulation; therefore, antibody markers against these antigens are expected to be highly sensitive and stable.

Identification of pre-onset and predictive markers for AIS and AMI will be critical not only for diagnosis but also treatment approaches. Thus, we performed SEREX screening using sera of patients with transient ischemic attack (TIA) which is a prodromal syndrome of AIS. Recent clinical studies have focused on early intervention benefits in patients with TIA to prevent the subsequent development of cerebral infarction [38]. However, the diagnosis of TIA is sometimes difficult because of the lack of objective evidences detected by various medical examinations such as magnetic resonance imaging (MRI), echo cardiogram, and Holter electrocardiogram. Likewise, non-ST segment elevation acute coronary syndrome (NSTEACS), which is an atherosclerotic heart disease, should be treated in the acute phase because it has the same mortality as AMI [39]. However, the diagnosis of NSTEACS is sometimes difficult in the emergency room compared to AMI, so we performed SEREX screening using sera of patients with NSTEACS aiming a convenient diagnosis. DnaJ heat shock protein family (Hsp40) member C2 (DNAJC2), an antigen commonly recognized by serum IgG antibodies from patients with TIA and NSTEACS, is thus a potentially useful biomarker for the detection of them.

## 2 Materials and methods

### 2.1 Ethical approval

This study was approved by the Ethical Review Board of the Graduate School of Medicine at Chiba University and the cooperating hospitals. All procedures were performed according to the principles of the Declaration of Helsinki. Recombinant DNA studies were approved by the Graduate School of Medicine at Chiba University and complied with the rules of the Japanese government. Written informed consent was obtained from all participants.

### 2.2 Sera from patients and healthy donors

All enrolled patients experienced ischemic stroke and were admitted to the hospital within two weeks from the onset of stroke. Individuals with no history of ischemic stroke were enrolled as healthy donors (HDs). HDs underwent medical checkups, including cerebral MRI. Patients with autoimmune diseases were excluded from the study. The study population was divided into independent screening and validation cohorts.

The screening cohort consisted of 20 Japanese patients with TIA. The validation cohort consisted of 621 patients and 285 HDs. In the validation cohort, 92 patients had TIAs, 464 had AISs, and 65 had chronic-phase cerebral infarctions (cCIs). Serum samples from the 20 patients with TIA in the screening cohort were not used in the validation cohort. Table 1 shows the clinical characteristics of the patients and HDs. To validate the relationship between antibody levels and AMI and DM, 128 patients and 128 age-matched HDs were selected. CKD patients in the Kumamoto cohort [40] were selected to validate the relationship between antibody levels and CKD.

**Table 1:** Baseline characteristics of subjects.

	Screening	Validation cohort			
	TIA (n = 20)	Stroke (n = 621)			HD (n = 285)
		cCI (n = 65)	AIS (n = 464)	TIA (n = 92)	
Age (years)	67.5 ± 19.1	73.3 ± 9.2∗∗	75.5 ± 11.5∗∗	70.2 ± 11.6∗∗	52.3 ± 11.7
Male gender	12 (60.0%)	48 (73.8%)	271 (58.4%)	55 (59.7%)	188 (65.9%)
Hypertension	13 (65.0%)	53 (81.5%)∗∗	335 (72.2%)∗∗	60 (65.2%)∗∗	57 (20.0%)
Diabetes	7 (35.0%)	22 (33.8%)∗∗	125 (26.9%)∗∗	26 (28.3%)∗∗	11 (3.9%)
Hyperlipidemia	8 (40.0%)	25 (38.5%)∗∗	122 (26.3%)∗∗	36 (39.1%)∗∗	40 (14.0%)
CVD	1 (5.0%)	2 (3.1%)∗∗	40 (8.6%)∗∗	5 (5.4%)∗∗	0 (0.0%)
Obesity (BMI ≥ 25)	5 (25.0%)	11 (16.9%)	127 (27.4%)	30 (32.6%)	88 (30.9%)
Smoking	11 (55.0%)	33 (50.8%)	228 (49.1%)	43 (46.7%)	132 (46.3%)

Data represents means (±SD) for continuous data and n (%) for categorical data.

∗∗*p* < 0.001 versus HD.

TIA, transient ischemic attack; AIS, acute ischemic stroke; HD, healthy donor; cCI, chronic cerebral infarction; CVD, cardiovascular disease.

Serum samples from patients with TIA, cCI, and AIS were obtained from Chiba Prefectural Sawara Hospital, Chiba Rosai Hospital, and Chiba Aoba Municipal Hospital. Sera of patients with AMI, DM, and CKD were obtained from Kyoto University Hospital, Chiba University Hospital, and the Kumamoto cohort, respectively. Sera of patients with NSTEACS were obtained from Chiba University Hospital. Sera of HDs were obtained from Chiba Prefectural Sawara Hospital, Higashi Funabashi Hospital, and Port Square Kashiwado Clinic. After collection, samples were centrifuged at 3,000 *g* for 10 min at room temperature and the supernatants were stored at −80 °C until use. Samples were not repeatedly thawed and frozen.

### 2.3 Clinical data

Patient data related to risk factors for atherosclerosis, including age, sex, hypertension, DM, hyperlipidemia, CVD, obesity, and smoking, were collected from clinical records. Hypertension was defined as a history of systolic blood pressure >140 mmHg, diastolic blood pressure >90 mmHg, or antihypertensive agent use. DM was defined as a history of DM diagnosed and/or treated with medication and/or fasting blood glucose ≥126 mg/dL. Hyperlipidemia was defined as a history of TC > 220 mg/dL, triglycerides >150 mg/dL, or lipid-lowering agent use. CVD was defined as a history of MI or angina pectoris. Patients were considered smokers if they smoked during the study period or had a history of smoking. Obesity was defined as a body mass index (BMI) ≥ 25. TIA was defined as a transient episode of neurological dysfunction caused by focal brain, spinal cord, or retinal ischemia, without acute infarction [41]. Patients presenting without persistent ST-segment elevation on ECG were defined as NSTEACS, including patients who experienced non-ST segment elevation myocardial infarction (NSTEMI) and patients with unstable angina with myocardial ischemia but no myocyte necrosis [42].

### 2.4 Screening by expression cloning

Immunoscreening was performed as previously described with modifications [25, 27, 28, 29, 34, 43]. A commercially available human aortic endothelial cell cDNA phage library (Uni-ZAP XR Premade Library, Stratagene, La Jolla, CA) was used to screen for clones that were immunoreactive against the sera of patients with TIA or UAP. Briefly, *Escherichia coli* (*E. coli*) XL1-Blue MRFʹ cells were infected with the Uni-ZAP XR phage. The expression of resident cDNA clones was induced after blotting infected bacteria onto nitrocellulose membranes (NitroBind, Osmonics, Minnetonka, MN) pretreated with 10 mM isopropyl-β-D-thiogalactoside (IPTG; Wako Pure Chemicals, Osaka, Japan) for 30 min. The membranes with bacterial proteins were washed three times with Tris-buffered saline [20 mM Tris-HCl (pH 7.5), 0.15 M NaCl] containing 0.05% Tween-20 (TBS-T). Then, nonspecific binding was blocked with 1% protease-free bovine serum albumin (Nacalai Tesque, Kyoto, Japan) in TBS-T for 1 h. The membranes were incubated overnight with 1:2000 dilutions of patient sera. After three washes with TBS-T, the membranes were incubated for 1 h with 1:5000-diluted alkaline phosphatase-conjugated goat anti-human IgG (Jackson ImmunResearch Laboratories, West Grove, PA). Positive reactions were visualized using a color development solution [100 mM Tris-HCl (pH 9.5), 100 mM NaCl, and 5 mM MgCl_2_] containing 0.15 mg/ml of 5-bromo-4-chloro-3-indolylphospate (Wako Pure Chemicals) and 0.3 mg/ml of nitro blue tetrazolium (Wako Pure Chemicals). Positive clones were recloned two more times to obtain monoclonality, as described previously [25, 27, 28, 29, 34, 43].

### 2.5 Sequence analysis of identified antigens

*In vivo* excision was used to convert monoclonal phage cDNA clones to pBluescript phagemids using ExAssist helper phage (Stratagene). After transforming with the phagemid, plasmid DNA was extracted from the *E. coli* SOLR strain. The cDNA inserts were sequenced and compared to public database sequences obtained from the National Center for Biotechnology Information (NCBI) (http://blast.ncbi.nlm.nih.gov/Blast.cgi/).

### 2.6 Construction of expression vectors

The cDNA sequences were recombined in the pGEX-4T vector (GE Healthcare, Pittsburgh, PA) to construct the glutathione-S-transferase (GST)-fused protein expression plasmids, as described previously [25, 27, 28, 29, 34]. The pBluescript plasmids with the cDNA inserts were digested with *EcoRI* and *XhoI* and separated via agarose gel electrophoresis. GenElute™ Minus EtBr Spin Columns (Merck, Darmstadt, Germany) was used to isolate the cDNA fragments, which were ligated into pGEX-4T-1 in frame using the Ligation-Convenience Kit (Nippon Gene, Toyama, Japan). ECOS™-competent *E. coli* BL-21 cells (Nippon Gene) were transformed with the ligation mixtures.

### 2.7 Purification of recombinant candidate proteins

Transformed *E. coli* BL-21 cells containing the pGEX-4T clones were cultured in 200 ml Luria broth and treated with 0.1 mM IPTG for 3 h. Cells were then harvested, washed with phosphate-buffered saline, and lysed by sonication in BugBuster Master Mix (Merck). The cell lysates were centrifuged at 13,000 *g* for 10 min at 4 °C. Recombinant GST and GST-DNAJC2 proteins were purified from the supernatants using glutathione-Sepharose column chromatography (GE Healthcare) and the purified proteins were concentrated using an Amicon Ultra-15 centrifugal filter device (Merck) [44].

### 2.8 Western blotting

GST and GST-fusion proteins (0.4 μg) were separated on sodium dodecyl sulfate-polyacrylamide gels. After transfer, the membranes were incubated with anti-GST antibodies (Rockland, Gilbertsville, PA) or sera from HDs or patients with TIA, AIS, NSTEACS, and AMI. After incubation with a horseradish peroxidase-conjugated secondary antibody, immunoreactivity was detected with Immobilon (Merck), as described previously [25, 27, 28, 43, 45].

### 2.9 AlphaLISA

Amplified luminescent proximity homogeneous assay-linked immunosorbent assay (AlphaLISA) was performed, as described previously [27, 28, 29, 30, 34, 35, 36, 37]. The assay was performed in 384-well microtiter plates (white opaque OptiPlate™, Perkin Elmer, Waltham, MA) containing 2.5 μl of sera (1:100 dilution) and 10 μg/ml GST or GST-fusion proteins in 2.5 μl of AlphaLISA buffer [25 mM HEPES (pH 7.4), 0.1% casein, 0.5% Triton X-100, 1 mg/ml dextran-500, and 0.05% proclin-300]. After incubating the reaction mix at room temperature for 6–8 h, anti-human IgG-conjugated acceptor beads (40 μg/ml in 2.5 μl) and glutathione-conjugated donor beads (40 μg/ml in 2.5 μl) were added and the samples were incubated for seven days at room temperature in the dark. Chemiluminescence was examined using an EnSpire Alpha microplate reader (Perkin Elmer). Specific reactions were calculated by subtracting the Alpha photon counts of GST controls from those of the GST-fusion proteins.

### 2.10 Statistical analyses

Mann–Whitney *U* or Kruskal–Wallis tests were utilized, as appropriate, for analysis of continuous variables. Categorical variables were analyzed with a Chi-square test. To identify the variables that classify participants based on a history of ischemic stroke, univariate and multivariate logistic regression analyses were performed. The cutoff values for antibody levels were determined to maximize the sum of sensitivity and specificity by receiver operating characteristic (ROC) curve analysis. All comparisons were planned, and the tests were two-tailed. A *p* value of less than 0.05 was considered statistically significant. Univariate and multivariate logistic regression and ROC curve analyses were performed using JMP Pro 13.0.0 software (SAS Institute Inc., Cary, NC). All other analyses were performed using GraphPad Prism 5 (GraphPad Software, La Jolla, CA).

## 3 Results

### 3.1 Screening by expression cloning

A total of 2 × 10^6^ cDNA clones were screened using sera of 20 patients with TIA and isolated 36 reactive clones. DNA sequence analysis and search for homologous sequences in an NCBI-accessible database indicated that these isolated clones comprised 18 independent genes, including two genes related to heat shock proteins: DnaJ heat shock protein family (Hsp40) member A1 (*DNAJA1*) and DnaJ heat shock protein family (Hsp40) member C2 (*DNAJC2*) (Table 2). DNAJC2 was also isolated by SEREX screening using sera of patients with NSTEACS, based on the coding sequence in base positions between 252 and 2117 shown in the database. The clone isolated by the TIA sera contained base positions between 176 and 925 corresponding to amino acid positions between 1 and 224, whereas that isolated by the NSTEACS sera involved base positions between 55 and 2167 which covered the full-length coding sequence. The epitope recognized by serum antibodies was estimated to exist within base positions between 176 and 925. This region was recombined into pGEX-4T-1 to successfully create the GST-fused DNAJC2 containing amino acid positions 1–224, which was purified by affinity chromatography and used as an antigen to examine serum antibody levels in subsequent experiments.

**Table 2:** Genes of candidate antigen in the screening.

Gene name	Full name (Homology)	Accession No.	CDS	Site of cloned region
*DNAJA1*	*DnaJ heat shock protein family (Hsp40) member A1 (DNAJA1), transcript variant 1*	NM_001539	192..1385	108..857
*DNAJC2*	*DnaJ heat shock protein family (Hsp40) member C2 (DNAJC2), transcript variant 1*	NM_014377	252..2117	176..925^a^ 55..2167^b^

a clone isolated by screening using sera of patients with TIA.

b clone isolated by screening using sera of patients with NSTEACS.

### 3.2 Western blots of purified antigens

**Figure 1:**
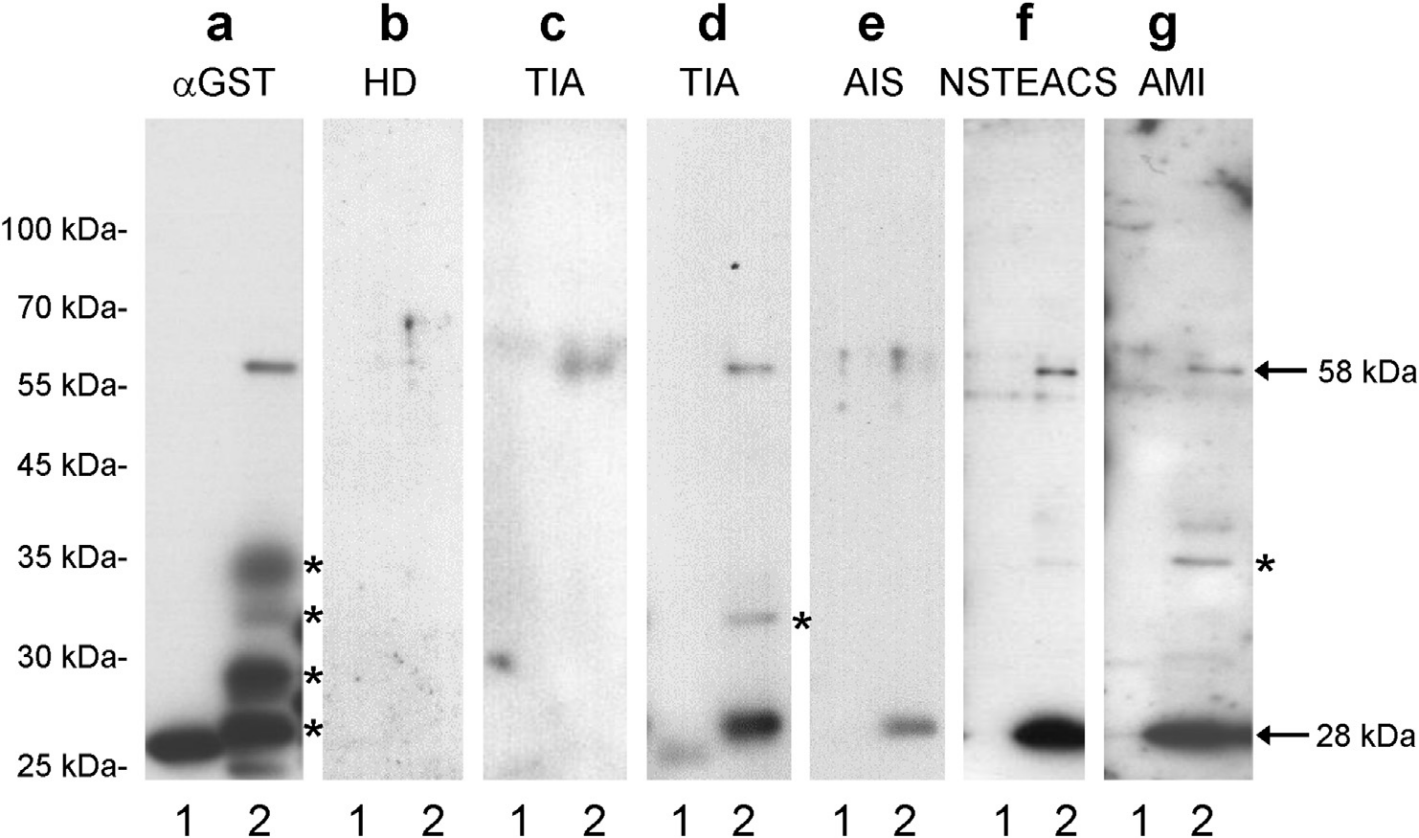
Western blot analysis. Glutathione-S-transferase (GST) (lane 1) and affinity-purified GST-tagged DnaJ heat shock protein family (Hsp40) member C2 (DNAJC2) (lane 2) proteins were separated on sodium dodecyl sulfate-polyacrylamide gels and blotted using an anti-GST antibody (**a**); the sera of healthy donors (HDs) (**b**); or the sera of patients with transient ischemic attack (TIA) (**c**, **d**), acute ischemic stroke (AIS) (**e**), non-ST segment elevation acute coronary syndrome (NSTEACS). (**f**), and acute myocardial infarction (AMI) (**g**). Arrows at 58 kDa and 28 kDa indicate GST-DNAJC2 and GST proteins, respectively. Asterisks indicate partially degraded proteins. Molecular weights are shown on the left. The full, non-adjusted image of Figure 1 is shown in the supplementary figure S1.

Next, the presence of anti-DNAJC2 antibody (DNAJC2-Ab) in the patient sera was confirmed by western blotting. GST-DNAJC2 and GST proteins were detected as 58-kDa and 28-kDa proteins, respectively, using the anti-GST antibody (Figure 1a). The molecular weight of the largest product was similar to that predicted by sequencing analysis including linker sequences. GST-DNAJC2 reacted with serum IgG antibodies of the patients with TIA, AIS, NSTEACS, and AMI but not with those from HDs (Fig. 1b–g). The GST protein showed very low, if any, reactivity against serum IgG from either the HDs or the patients.

### 3.3 Validation of elevated DNAJC2-Ab levels in stroke patients

**Figure 2:**
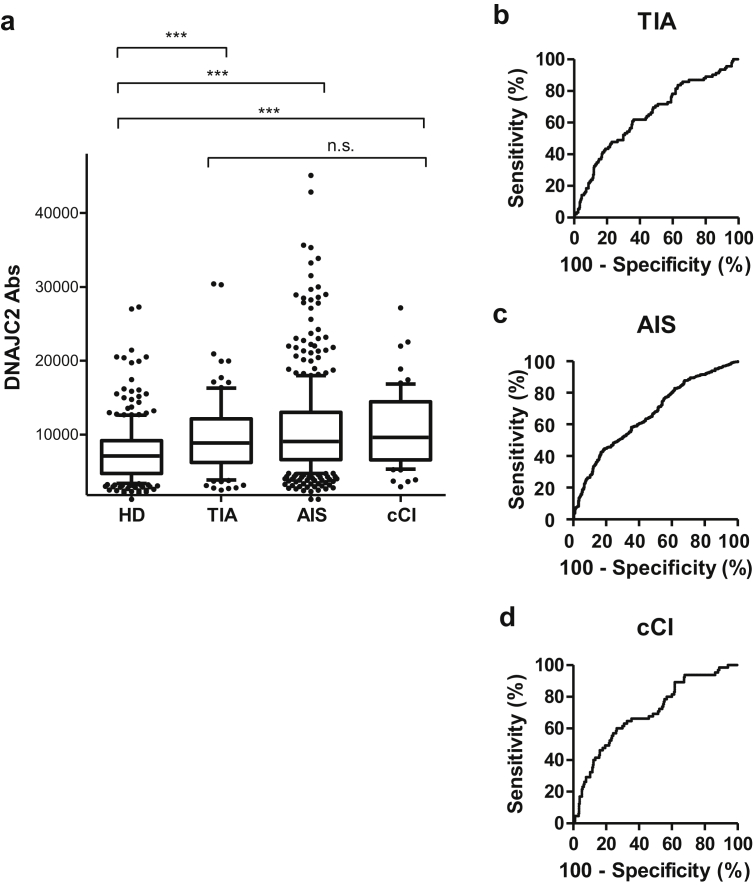
Serum levels of DNAJC2 antibodies (DNAJC2-Abs) in stroke patients examined by amplified luminescent proximity homogeneous assay-linked immunosorbent assay (AlphaLISA) in the validation cohort. The DNAJC2-Ab levels measured as Alpha photon counts were compared between the HDs and the patients with TIA, AIS, or chronic-phase cerebral infarction (cCI) in box-whisker plots displaying the 10^th^, 20^th^, 50^th^, 80^th^, and 90^th^ percentiles (**a**). ∗∗∗*p* < 0.001 by the Mann–Whitney *U* test with type I error adjustment using the Bonferroni procedure, not significant (n.s.), *p* = 1 by the Kruskal–Wallis test with type I error adjustment using the Bonferroni procedure. Receiver operating characteristic curve (ROC) analysis was performed to assess the ability of DNAJC2-Abs to detect TIA (**b**), AIS (**c**), and chronic cerebral infarction (cCI) (**d**). Table 3 summarizes areas under the curves (AUCs), 95% confidence intervals (CIs), cutoff values, sensitivity, specificity, and *p* values calculated using ROC analysis.

The elevation of the DNAJC2-Ab levels in stroke patients was validated in the independent validation cohort (n = 906; Table 1). AlphaLISA revealed significantly higher DNAJC2-Ab levels in the patients with TIA (*p* < 0.001), AIS (*p* < 0.001), and cCI (*p* < 0.001), compared with the HDs (Figure 2). Briefly, the mean DNAJC2-Ab levels (Alpha photon counts; ± standard deviation) in patients with TIA, AIS, and cCI and HDs were 9903 ± 5195, 10825 ± 7774, 10701 ± 4961, and 7766 ± 5003, respectively. However, no significant differences in DNAJC2-Ab levels were observed among the patients with TIA, AIS, and cCI. The ROC analysis showed that the area under the curve (AUC) values for TIA, AIS, and cCI were 0.6477, 0.6619, and 0.6987, respectively (Figure 2b–d, Table 3). The sensitivity and a specificity for AIS were 44.83% and 81.05%, respectively.

**Table 3:** Summary of receiver operating characteristic (ROC) curve analysis.

	TIA	AIS	cCI	AMI	DM	Type 1 CKD	Type 2 CKD	Type 3 CKD
AUC∗	0.6477	0.6619	0.6987	0.6714	0.6765	0.8182	0.8232	0.7305
95% CI	0.5814–0.7140	0.6225–0.7013	0.6278–0.7697	0.6053–0.7376	0.6112–0.7419	0.7605–0.8759	0.7432–0.9031	0.6597–0.8013
Cutoff value	8,193	9,836	9,102	23,188	25,354	9,973	12,374	10,878
Sensitivity (%)	61.96	44.83	60.00	43.75	36.72	91.72	75.00	72.36
Specificity (%)	63.86	81.05	73.68	86.72	95.31	57.32	78.05	65.85
*p* value	<0.0001	<0.0001	<0.0001	<0.0001	<0.0001	<0.0001	<0.0001	<0.0001

∗Area under the curve (AUC) values, 95% confidence interval (CI), cutoff values, sensitivity (%), specificity (%), and *p* values calculated by ROC analysis are shown.

### 3.4 Association between DNAJC2-Ab levels and other clinical parameters in stroke patients

**Figure 3:**
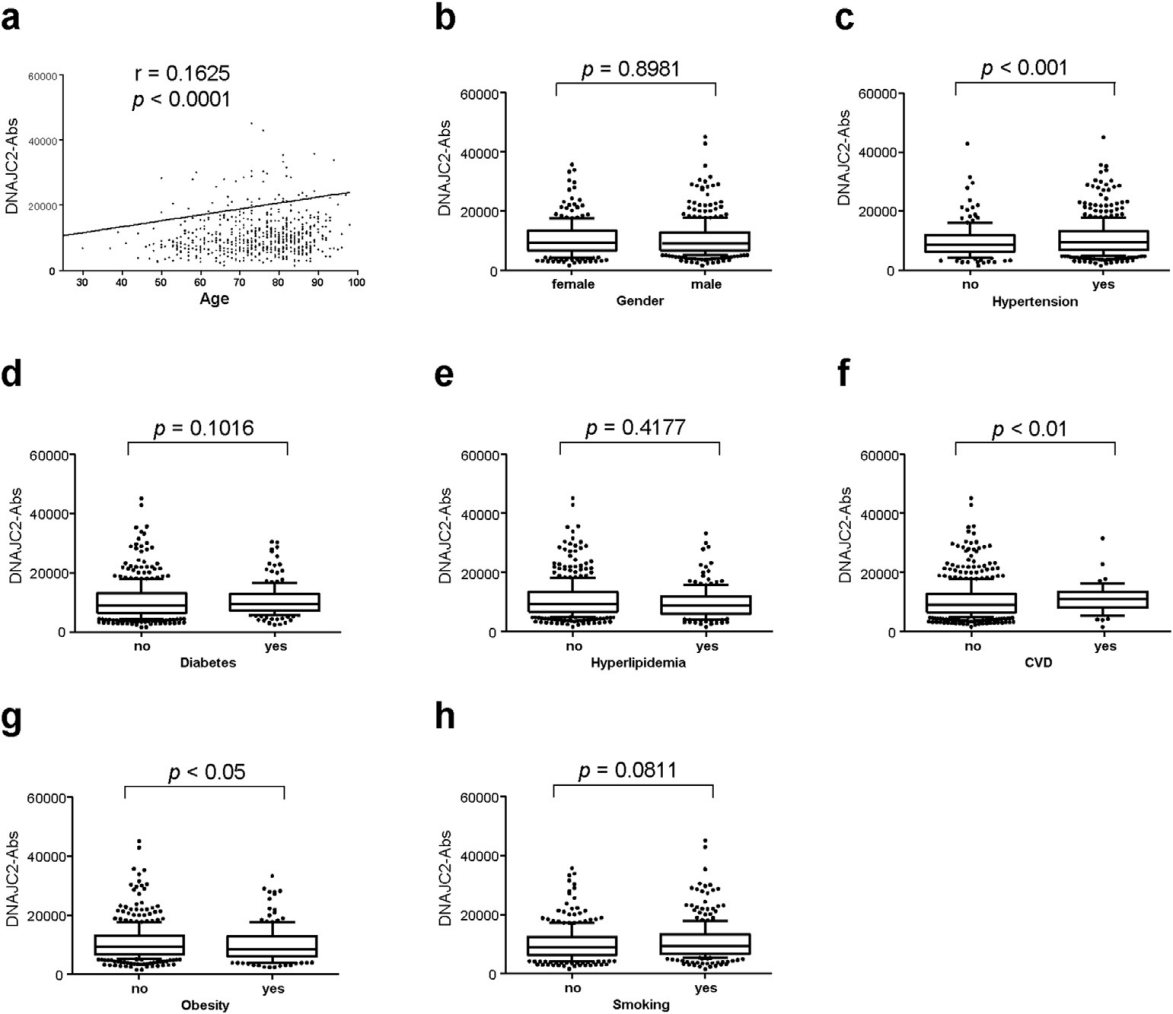
Association between DNAJC2-Ab levels and other clinical parameters in stroke patients. Correlations between DNAJC2-Ab levels and age (**a**), sex (**b**), hypertension (**c**), diabetes mellitus (DM) (**d**), hyperlipidemia (**e**), cardiovascular disease (CVD) (**f**), obesity (body mass index ≥25) (**g**), and smoking (**h**) were examined using Spearman's correlation analysis (**a**) or the Mann–Whitney *U* test (**b**–**h**).

Next, correlations between DNAJC2-Ab levels and clinical parameters were examined in the validation cohort. A weak association was observed between DNAJC2-Ab levels and age (r = 0.1625, *p* < 0.0001) and obesity (*p* = 0.443). The association was stronger in patients with CVD than those without the disease (*p* = 0.0011). Additionally, a strong association was observed between DNAJC2-Ab levels and hypertension (*p* < 0.0001) (Figure 3), whereas significant correlations were not observed between DNAJC2-Ab levels and other parameters, including sex, DM, hyperlipidemia, and smoking.

### 3.5 Association between stroke and clinical parameters including DNJC2-Ab levels

Table 4 shows the results of the univariate and multivariate logistic regression analyses. Using the DNAJC2-Ab cutoff value of 9837, univariate logistic regression analysis revealed that elevation in DNAJC2-Ab levels was associated with an increased risk of stroke [odds ratio (OR) 3.41, 95% confidence interval (CI): 2.44–4.76, *p* < 0.0001]. Multivariate logistic regression analysis revealed that elevation in DNAJC2-Ab levels was an independent predictor of stroke (OR 2.14, 95% CI: 1.39–3.30, *p* = 0.0005). The predictive value of DNAJC2-Ab for stroke was not inferior to those of known risk factors of stroke including age (OR 10.8, 95% CI: 7.26–16.1, *p* < 0.0001), hypertension (OR 4.96, 95% CI: 3.32–7.41, *p* < 0.0001), and DM (OR 5.71, 95% CI: 2.73–11.9, *p* < 0.0001).

**Table 4:** Logistic regression of predictive factors for AIS (n = 906; no. of events = 621).

	Univariate analysis			Multivariate analysis		
	OR	95% CI	*p* value	OR	95% CI	*p* value
Age (≥60)	18.9	13.2–26.9	<0.0001	10.8	7.26–16.1	<0.0001
Male	0.77	0.58–1.03	0.0835			
HT	10.4	7.38–14.5	<0.0001	4.96	3.32–7.41	<0.0001
DM	9.62	5.13–18.0	<0.0001	5.71	2.73–11.9	<0.0001
HL	2.56	1.76–3.73	<0.0001	1.00	0.61–1.64	0.9991
CVD	11.6	2.79–48.0	0.0007	2.37	0.54–10.5	0.2560
Obesity (BMI ≥ 25)	0.87	0.64–1.18	0.3767			
Smoking	1.13	0.85–1.49	0.4104			
DNAJC2 (>9837)∗	3.41	2.44–4.76	<0.0001	2.14	1.39–3.30	0.0005

HT, hypertension; DM, diabetes mellitus; HL, hyperlipidemia; CVD, cardiovascular disease; OR, odds ratio, 95% CI, 95% confidence interval.

∗ DNAJC2, elevated DNAJC2-Ab levels. DNAJC2-Ab cutoff was 9837 based on ROC curve analysis.

### 3.6 Association between TIA and clinical parameters including DNJC2-Ab levels

Next, the association of TIA with clinical parameters was examined using the DNAJC2-Ab cutoff value of 9837 for predicting TIA. Table 5 shows the results of the univariate and multivariate logistic regression analyses. Univariate logistic regression analysis revealed that elevation in DNAJC2-Ab levels was associated with an increased risk of TIA (OR 3.22, 95% CI: 1.94–5.34, *p* < 0.0001). Multivariate logistic regression analysis revealed that elevated in DNAJC2-Ab levels was an independent predictor of TIA (OR 2.54, 95% CI: 1.36–4.74, *p* = 0.0034). The predictive value of DNAJC2-Ab for TIA was not inferior to those of known risk factors of TIA, including age (OR 5.16, 95% CI: 2.75–9.66, *p* < 0.0001), hypertension (OR 3.38, 95% CI: 1.86–6.14, *p* < 0.0001), and DM (OR 4.15, 95% CI: 1.66–10.4, *p* = 0.0023).

**Table 5:** Logistic regression of predictive factors for TIA (n = 377; no. of events = 92).

	Univariate analysis			Multivariate analysis		
	OR	95% CI	*p* value	OR	95% CI	*p* value
Age (≥60)	9.97	5.65–17.6	<0.0001	5.16	2.75–9.66	<0.0001
Male	0.74	0.46–1.21	0.2304			
HT	7.50	4.47–12.6	<0.0001	3.38	1.86–6.14	<0.0001
DM	9.81	4.61–20.9	<0.0001	4.15	1.66–10.4	0.0023
HL	3.94	2.30–6.73	<0.0001	2.03	1.05–3.92	0.0342
CVD	8.13	1.55–42.7	0.0132	1.22	0.20–7.51	0.8297
Obesity (BMI ≥ 25)	1.13	0.68–1.88	0.6301			
Smoking	1.01	0.63–1.62	0.9653			
DNAJC2 (>9837)∗	3.22	1.94–5.34	<0.0001	2.54	1.36–4.74	0.0034

HT, hypertension; DM, diabetes mellitus; HL, hyperlipidemia; CVD, cardiovascular disease; OR, odds ratio, 95% CI, 95% confidence interval.

∗ DNAJC2, elevated DNAJC2-Ab levels. DNAJC2-Ab cutoff was 9837 based on ROC curve analysis.

### 3.7 Association of serum DNAJC2-Ab levels with AMI and DM

**Figure 4:**
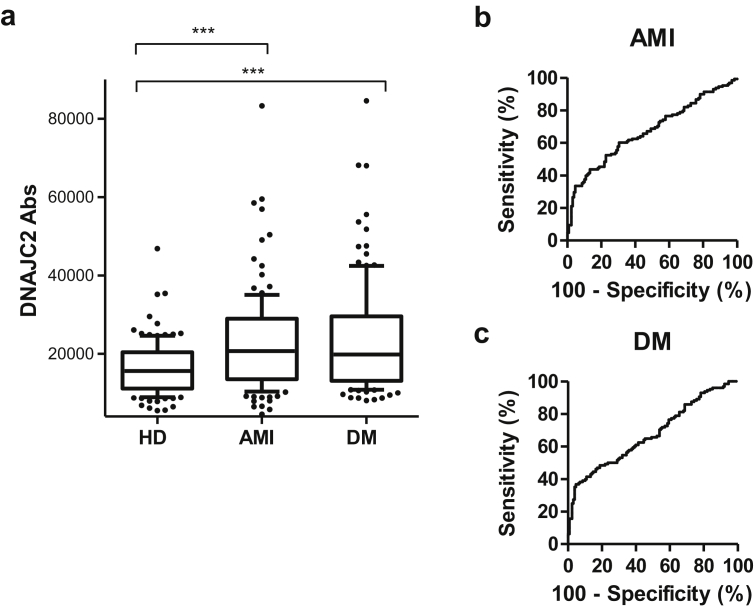
Association between DNAJC2-Ab levels and other atherosclerotic diseases including AMI and DM. (**a**) Serum DNAJC2-Ab levels of the HDs and the patients with AMI or DM determined by AlphaLISA are shown as box-whisker plots, as described in the legend of Figure 2. The mean ages (±standard deviation) of the HDs and the patients with AMI and DM were 58.29 ± 5.63, 58.28 ± 8.5, and 58.37 ± 9.11 years, respectively. Results of the ROC analysis of DNAJC2-Abs to detect AMI (**b**), and DM (**c**) are also shown. ∗∗∗*p* < 0.001 by the Mann–Whitney *U* test with type I error adjustment using the Bonferroni procedure.

Furthermore, the correlations between DNAJC2-Ab levels and AMI and DM were evaluated in the validation cohort using the sera of 128 patients and 128 age-matched HDs. AlphaLISA revealed significantly higher DNAJC2-Ab levels in the patients with AMI (*p* < 0.001) and DM (*p* < 0.001) compared to those in HDs (Figure 4a). ROC analysis revealed that the AUC values for AMI and DM were 0.6714 and 0.6765, respectively, which were similar to those of TIA, AIS, and cCI (Figure 4b, c, Table 3).

### 3.8 Association of serum DNAJC2-Ab levels with CKD

**Figure 5:**
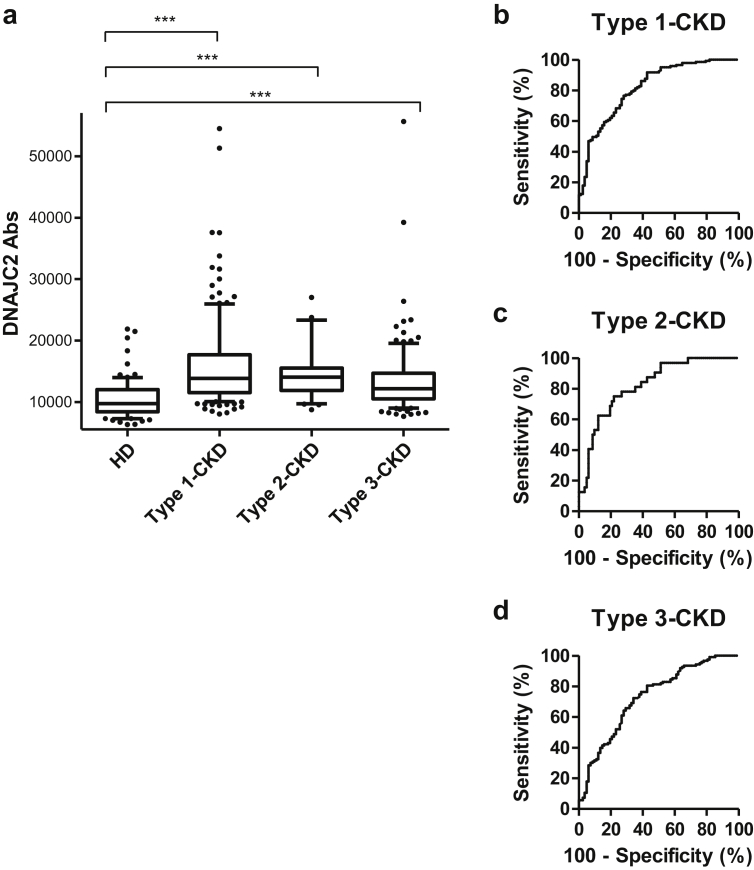
Association between DNAJC2-Ab levels and chronic kidney disease (CKD). (**a**) Serum DNAJC2-Ab levels of the HDs and the patients with CKD are shown as box-whisker plots, as described in the legend of Figure 2. CKD patients were divided into three groups: type 1, diabetic kidney disease; type 2, nephrosclerosis; and type 3, glomerulonephritis. The mean age (±standard deviation) of the HDs and the patients with type 1, 2, and 3 CKD were 45.82 ± 11.66, 65.78 ± 10.28, 75.97 ± 9.94, and 66.05 ± 14.60 years, respectively. Results of the ROC analysis of DNAJC2-Abs to detect type 1, 2, and 3 CKD (**b**–**d**) are also shown. ∗∗∗*p* < 0.001 by the Mann–Whitney *U* test with type I error adjustment using the Bonferroni procedure.

Finally, the correlations between DNAJC2-Ab levels and CKD were elucidated in the second validation cohort comprising 300 CKD patients and 82 HDs. The CKD patients were divided into three groups based on disease etiology: type 1, diabetic kidney disease; type 2, nephrosclerosis; and type 3, glomerulonephritis. Of the 300 CKD patients, 145, 32, and 123 had type 1, 2, and 3 CKD, respectively. AlphaLISA revealed significantly higher DNAJC2-Ab levels in the patients with CKD type 1 (*p* < 0.001), type 2 (*p* < 0.001), and type 3 (*p* < 0.001), compared with the HDs (Figure 4b). The AUC values for type 1, 2, and 3 CKD were 0.8182, 0.8232, and 0.7305, respectively, which were higher than those for TIA, AIS, cCI, AMI, and DM (Figure 5b–d, Table 3).

## 4 Discussion

The major finding of the current study was the elevated levels of DNAJC2-Ab in patients with atherosclerotic diseases, including stroke, AMI, DM, and CKD (Figures 2, 4, and 5, Table 3), which suggests that DNAJC2-Ab is potentially useful to evaluate the progress of atherosclerosis. In fact, DNAJC2 was screened using not only the sera of TIA patients but also those of the NSTEACS patients. The results were corroborated by western blotting (Figure 1). Furthermore, the elevation in DNAJC2-Ab levels was an independent predictor of stroke and TIA (Tables 4 and 5), suggesting that DNAJC2-Ab could discriminate atherosclerosis leading to stroke.

The isolated cDNA clones of DNAJC2 contained the full-length coding sequence and the 5′-terminal fragment, a region that is highly conserved among the DnaJ family members [46]. Similarly, the 5′-terminal fragment of DNAJA1 cDNA was also isolated by SEREX screening using the serum of a patient with TIA (Table 2). Therefore, serum IgG in these patients may also recognize other DnaJ family members.

Hsps comprise several groups based on their molecular weights: small Hsps, Hsp40, Hsp60, Hsp70, and Hsp90 [47]. An example of the Hsp40 family member is DnaJ with a molecular weight of 41 kDa [48, 49]. DNAJC2, a member of the DnaJ Hsp40 protein family, is a molecular chaperone that is expressed in a wide variety of organisms from bacteria to humans [50, 51]. Thus, anti-DnaJ antibody might be initially produced as a response to bacterial infection as suggested previously [52], and serum DnaJ antibody levels might be elevated due to the repeated release of the DnaJ family proteins, which are highly expressed in the atherosclerotic plaques, during the progression of atherosclerosis [53].

Previous studies suggested Hsps as major autoantigens involved in the pathogenesis of atherosclerosis [17, 18, 54], although their molecular roles in atherogenesis remain to be elucidated. Hsp70 was shown to protect endothelial cells from cytotoxic stress [55], whereas Hsp60 was able to stimulate monocyte and T cell adhesion to aortic endothelium, leading to plaque formation [56]. Hsp70/100 can protect methionine synthase activity, which suppresses atheromatous plaque expansion [57]. Furthermore, DnaJ acts as a cochaperone for Hsp70, and, thus, may have a role in atherogenesis.

The serum DNAJC2-Ab levels were higher in the patients with TIA, AIS, cCI, AMI, DM, and CKD than those in HDs (Figures 2, 4, and 5); the AUC values were highest in the CKD sera (Table 3). The most prominent association with DNAJC2 Abs was observed with type 1 CKD (diabetic kidney disease) and type 2 CKD (nephrosclerosis). Therefore, the primary cause of the elevation in DNAJC2-Abs may be attributable to kidney failure, which could induce atheromatous plaque development. DM can indirectly induce atherosclerosis via kidney failure such as that observed in type 1 CKD. Consequently, TIA, AIS, cCI, and AMI caused by atherosclerosis were associated with the DNAJC2 Ab levels.

The onset of AIS and AMI can lead to the production of numerous antigenic proteins. However, autoantibodies to such antigenic proteins cannot be detected soon after the onset of disease. The serum samples of AIS and AMI were collected within two weeks (one week in the majority of the patients) after the onset and exhibited higher DNAJC2-Ab levels than those in HDs (Figures 2 and 4). Thus, DNAJC2-Abs were present before the onset, suggesting the possible utility of DNAJC2-Ab as a predictive marker. Consistently, DNAJC2-Ab levels were also elevated in patients with TIA (Figure 2, Table 5) which is a potential prodromal syndrome of AIS. Further cohort longitudinal studies are necessary to confirm the usefulness of DNAJC2-Ab marker for prediction of the onset.

This study has several limitations. First of all, patient and control samples are in low volume. Antibody markers for atherosclerotic disease have odds ratios similar to well-known clinical risk factors such as age, hypertension, diabetes. Moreover, the diagnostic value of DNAJC2 alone was weak (sensitivity and specificity were 44.83% and 81.05%, respectively). It is possible that the diagnostic value is improved by a combination of the DNAJC2-Ab levels and clinical risk factors, including age, hypertension, and diabetes, which were independent predictive factors for stroke in the multivariate logistic regression analysis (Table 4). In fact, the positive predictive values with the combination of DNAJC2-Ab and clinical risk factors were higher than those with clinical risk factors alone (Table 6). Thus we believed that DNAJC2-Ab by simple blood test contributes to prediction of the onset of arteriosclerotic diseases as one risk factor.

**Table 6:** Validation of predictive factors for stroke (n = 906; no. of events = 621).

	Clinical risk factor			Clinical risk factor + DNAJC2 (>9837)∗		
	Stroke (+)	Stroke (-)	PPV	Stroke (+)	Stroke (-)	PPV
Age (≥60)	545	79	87.3%	258	17	93.8%
HT	448	57	88.7%	212	15	93.4%
DM	173	11	94.0%	82	1	98.8%
Age (≥60) + HT	406	28	93.5%	199	7	96.6%
Age (≥60) + DM	154	5	96.9%	76	1	98.7%
HT + DM	140	5	96.6%	69	0	100%
Age (≥60) + HT + DM	128	2	98.5%	65	0	100%

HT, hypertension; DM, diabetes mellitus; PPV, positive predictive value.

∗ DNAJC2, elevated DNAJC2-Ab levels. DNAJC2-Ab cutoff was 9837 based on ROC curve analysis.

In conclusion, serum DNAJC2-Ab levels were elevated in patients with a variety of atherosclerotic diseases, compared with HDs, and therefore, they can serve as a biomarker for stroke or myocardial infarction caused by advanced atherosclerosis.

## Declarations

### Author contribution statement

Yoichi Yoshida: Conceived and designed the experiments; Performed the experiments; Analyzed and interpreted the data; Wrote the paper.

Xiao-Meng Zhang, Hao Wang, Katsuro Iwase, Go Tomiyoshi, Rika Nakamura, Natsuko Shinmen: Performed the experiments.

Toshio Machida, Takaki Hiwasa: Conceived and designed the experiments; Wrote the paper.

Seiichiro Mine, Ikuo Kamitsukasa, Takeshi Wada, Akiyo Aotsuka, Hirotaka Takizawa, Koichi Kashiwado, Hideo Shin, Mikiko Ohno, Kenichiro Kitamura: Contributed reagents, materials, analysis tools or data.

Eiichi Kobayashi, Hideyuki Kuroda, Yasuo Iwadate: Conceived and designed the experiments.

Tomoo Matsutani: Analyzed and interpreted the data; Wrote the paper.

Akihiko Adachi, Yuichi Akaogi, Junichiro Shimada, Eiichiro Nishi, Minoru Takemoto, Koutaro Yokote: Analyzed and interpreted the data.

### Funding statement

This work was supported, in part, by research grants from the 10.13039/100009619Japan Agency for Medical Research and Development (AMED) (Practical Research Project for Life-Style related Diseases including Cardiovascular Diseases and Diabetes Mellitus), 10.13039/501100002241Japan Science and Technology Agency, and JSPS KAKENHI Grant Number 20K17953, 19K09451, 17K16626, 15K01842, 20H03449, 17K09575.

### Competing interest statement

The authors declare the following conflict of interests: This work was performed in collaboration with Fujikura Kasei Co., Ltd. GT, RN, NS, and HK are employees of Fujikura Kasei Co., Ltd.

### Additional information

No additional information is available for this paper.
