# Supplementary material for: Elevated levels of autoantibodies against DNAJC2 in sera of patients with atherosclerotic diseases
Source: Heliyon. 2020 Aug 19;6(8):e04661. doi: 10.1016/j.heliyon.2020.e04661 (PMC7452465; doi:10.1016/j.heliyon.2020.e04661)
Supplement: supplementary figure S1 [file mmc1.pptx]

## Slide 1
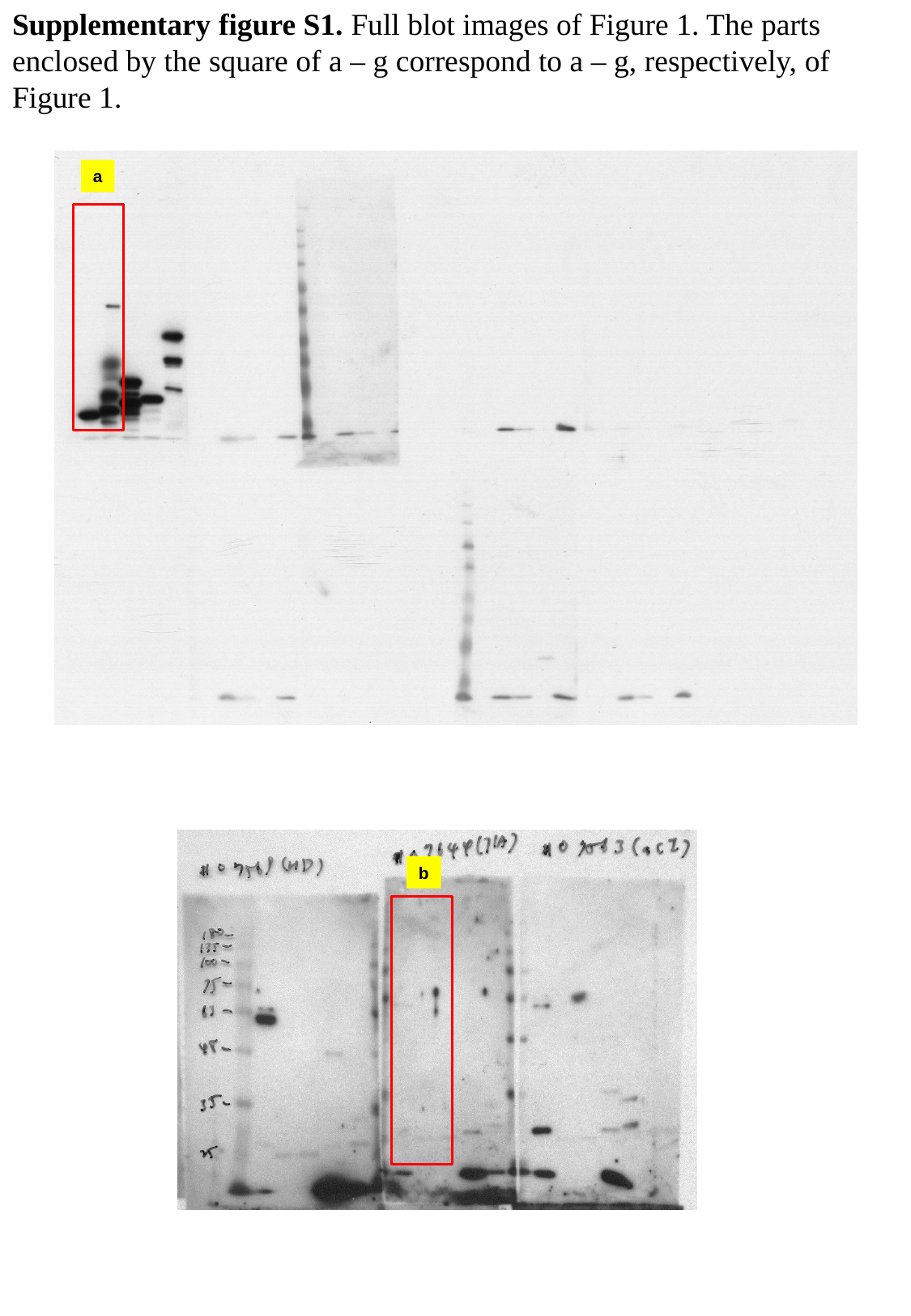

Supplementary figure S1. Full blot images of Figure 1. The parts enclosed by the square of a – g correspond to a – g, respectively, of Figure 1.
a
b

## Slide 2
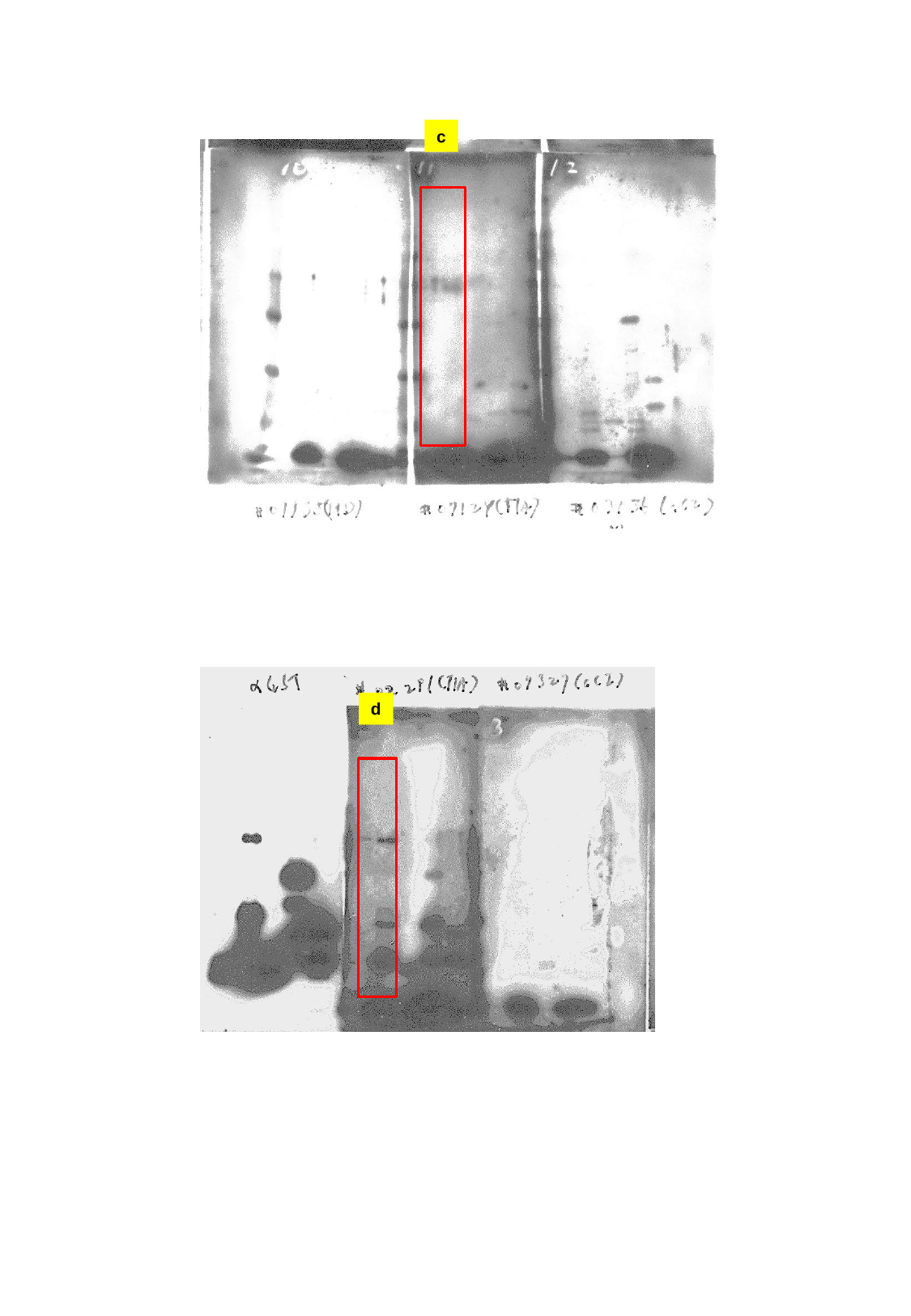

c
d

## Slide 3
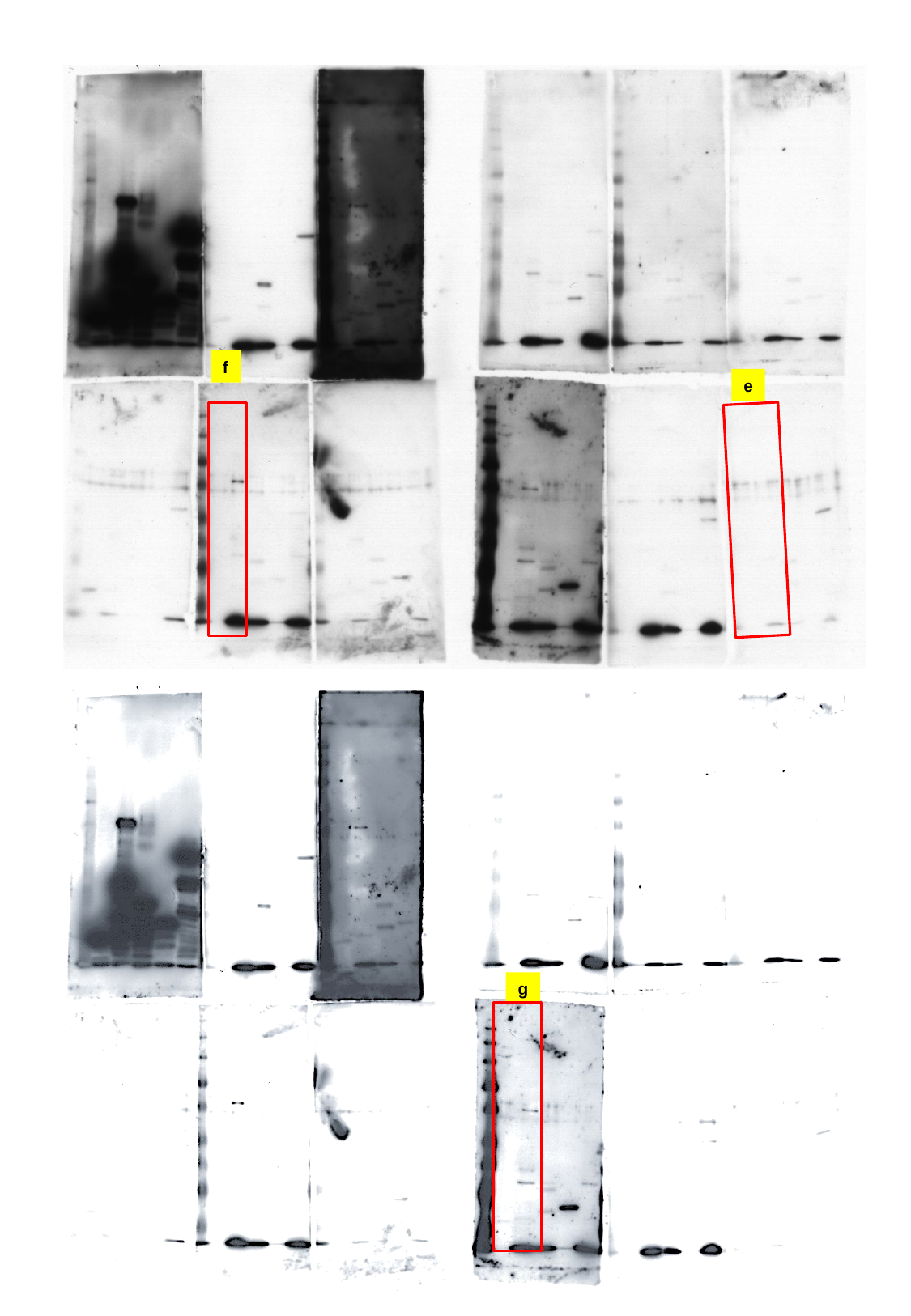

f
e
g
